# Supplementary material for: BRAF status modulates Interelukin-8 expression through a CHOP-dependent mechanism in colorectal cancer
Source: Commun Biol. 2020 Oct 1;3:546. doi: 10.1038/s42003-020-01263-y (PMC7530707; doi:10.1038/s42003-020-01263-y)
Supplement: Supplementary file 1 — Supplementary Information [file 42003_2020_1263_MOESM1_ESM.pdf]

# Contexts


| | | |
|---|---|---|
| A. | Supplementary Fig. 1 | 2 |
| B. | Supplementary Fig. 2 | 3 |
| C. | Supplementary Fig. 3 | 4 |
| D. | Supplementary Fig. 4 | 5 |
| E. | Supplementary Fig. 5 | 6 |
| F. | Supplementary Fig. 6 | 7 |
| G. | Supplementary Fig. 7 | 8 |
| H. | Supplementary Fig. 8 | 9 |
| I. | Supplementary Fig. 9 | 11 |
| J. | Supplementary Fig. 10 | 13 |
| K. | Supplementary Fig. 11, Original images for Western Blot | 14 |
| L. | Supplementary Fig. 12 | 15 |
| M. | Supplementary Table 1. | 16 |

# A. Supplementary Fig. 1

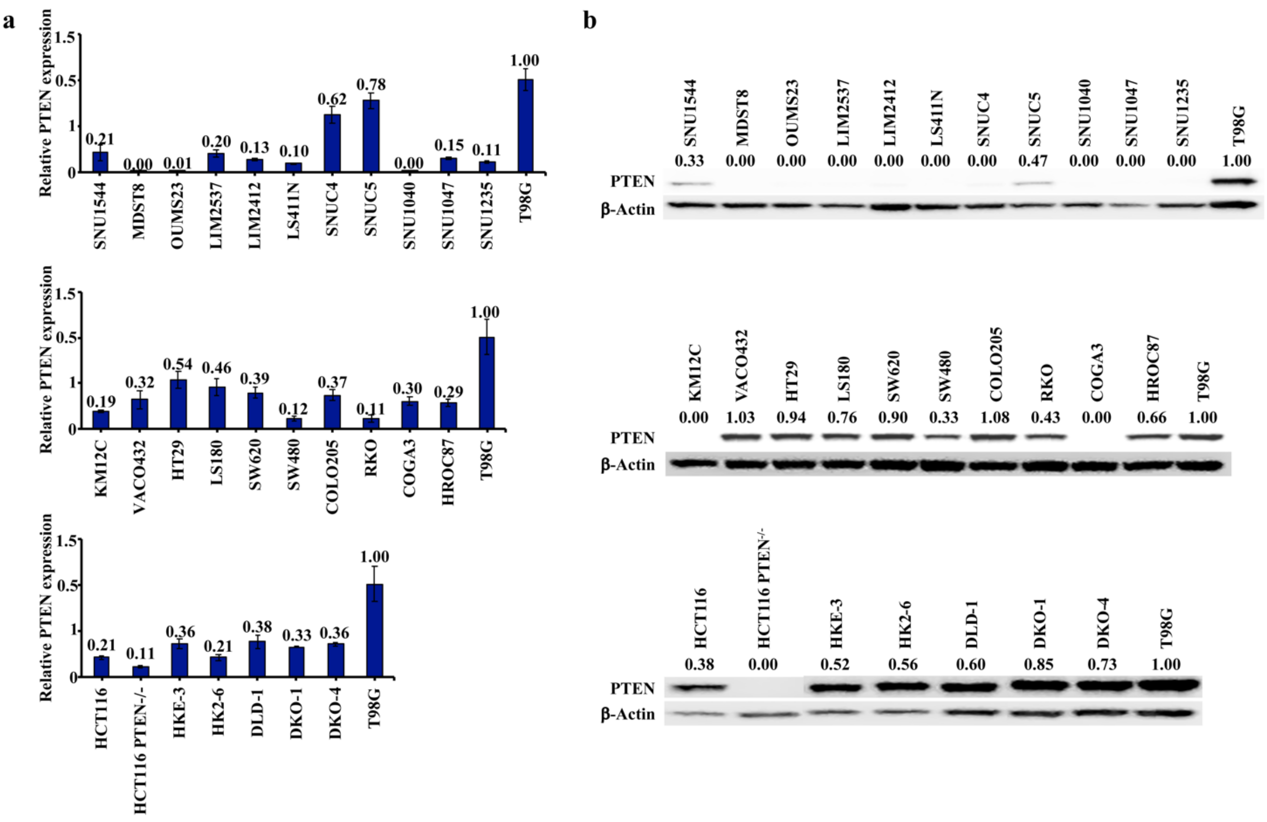


**PTEN characterization in a panel of 28 CRC cell lines.** Characterization of 28 CRC cell lines for both PTEN mRNA (**a**) and protein levels (**b**), by RT-qPCR and WB, respectively. T98G were used as a positive control for both PTEN mRNA and protein expression. For PTEN mRNA analysis, results were evaluated as ΔΔct of PTEN mRNA relative to RPL19 and expressed as the ratio, assuming the levels in T98G positive control cells as 1.0. Ratio of PTEN antibody/β-Actin for each individual sample was compared with the positive control T98G (1.00).

B. Supplementary Fig. 2

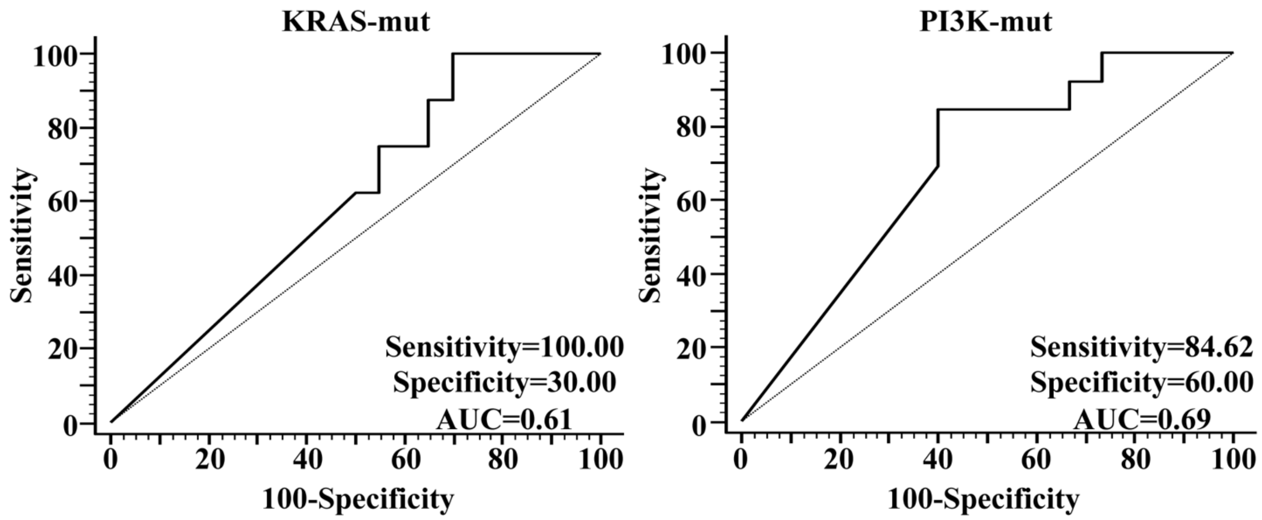


**IL-8 analysis and KRAS/PI3K mutational status.** 28 CRC cell lines were analyzed for their relative IL-8 expression, by IL-8 ELISA assay; IL-8 levels were then correlated with their genetic background of *KRAS* and *PI3K*.

C. Supplementary Fig. 3

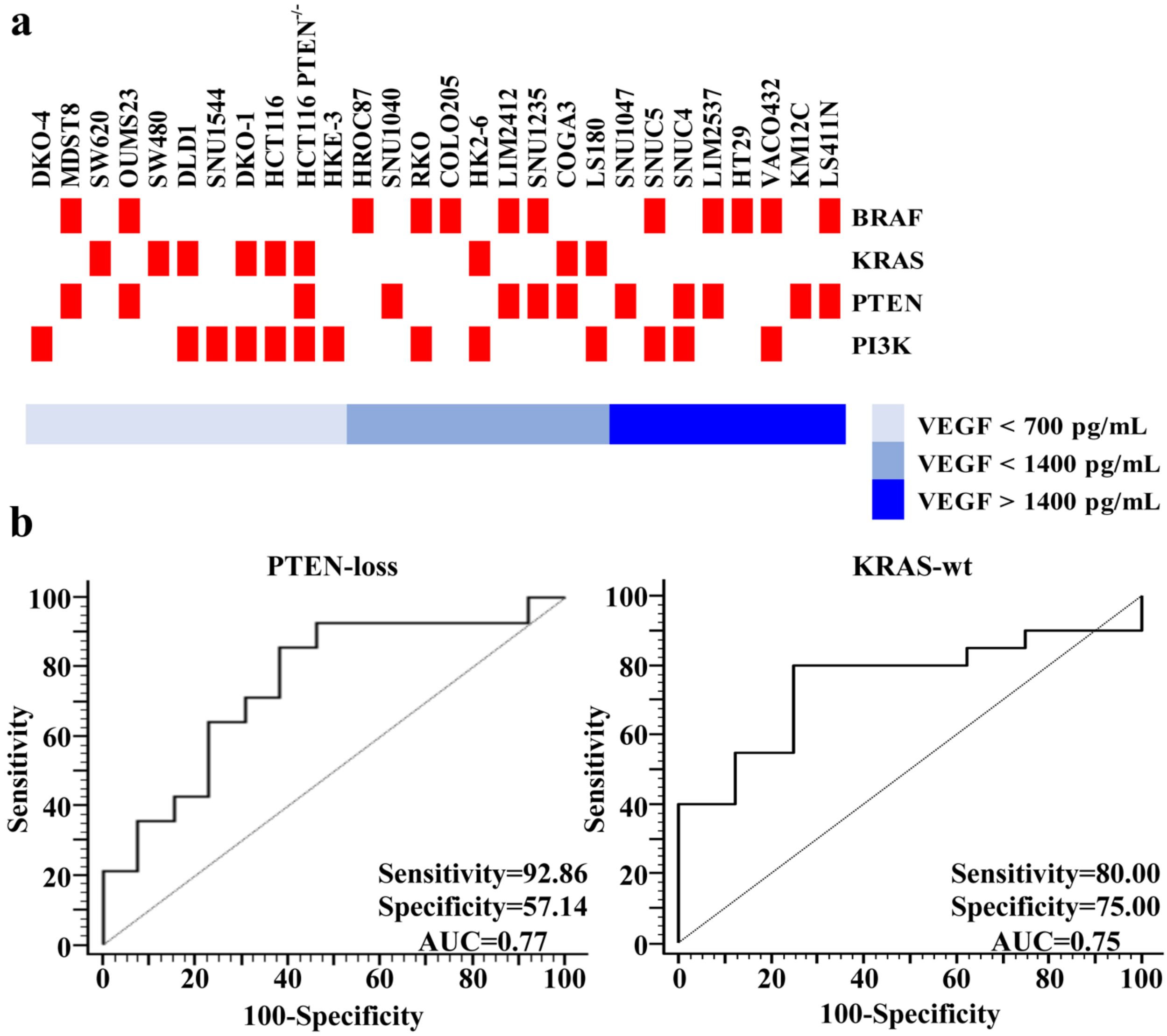


**VEGF expression in a panel of 28 CRC cell lines.** 28 CRC cell lines analyzed for their relative VEGF expression (shade of blue) and their genetic background of *BRAF*, *KRAS* and *PI3K* or PTEN protein lack expression (reported in red) (**a**). VEGF levels were measured by VEGF ELISA assay and the results were expressed as pg/mL for $1x10^6$ cells. Predicting high levels of VEGF expression according to PTEN and *KRAS* status (**b**).

D. Supplementary Fig. 4

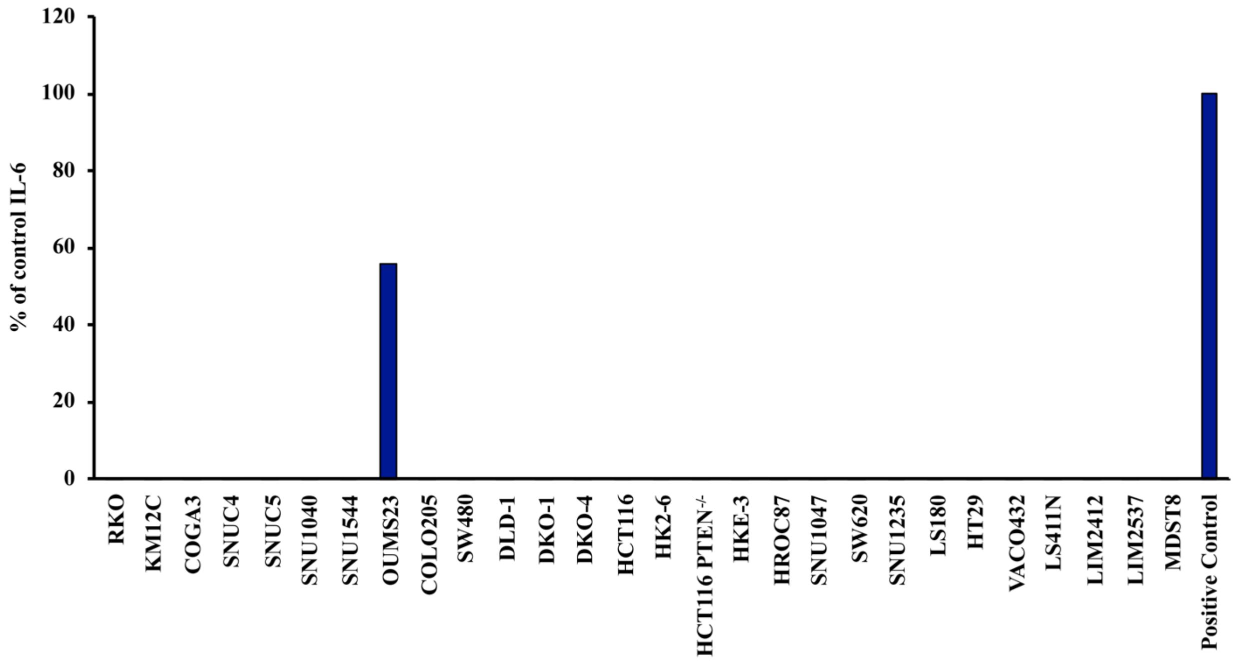


**IL-6 expression in a panel of 28 CRC cell lines.** 28 CRC cell lines analyzed for their relative IL-6 expression. IL-6 levels were measured by IL-6 ELISA assay; the percentage of IL-6 was obtained from pg/mL assuming the levels in control cells as 100%.

E. Supplementary Fig. 5

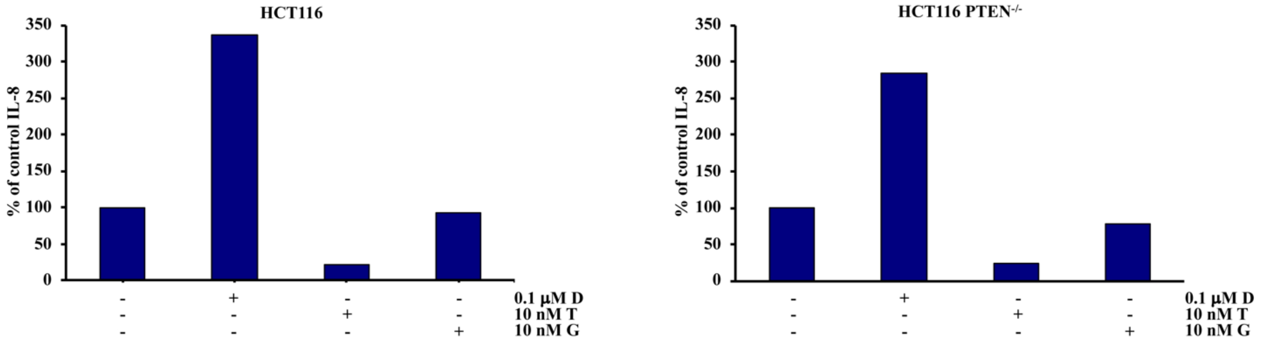


**Effects of drug inhibitors on IL-8 expression in isogenic HCT116 model.** X-MAN™ isogenic HCT116 cell lines (HCT116 and HCT116 PTEN$^{-/-}$) were treated with fixed doses of dabrafenib (D), trametinib (T) or gedatolisib (G), as indicated. IL-8 expression was measured after 24 hours of treatment, by IL-8 ELISA assay; the percentage of IL-8 was obtained from pg/mL assuming the levels in control cells as 100%. Results of a representative experiment out of three independent experiments performed are shown.

F. Supplementary Fig. 6

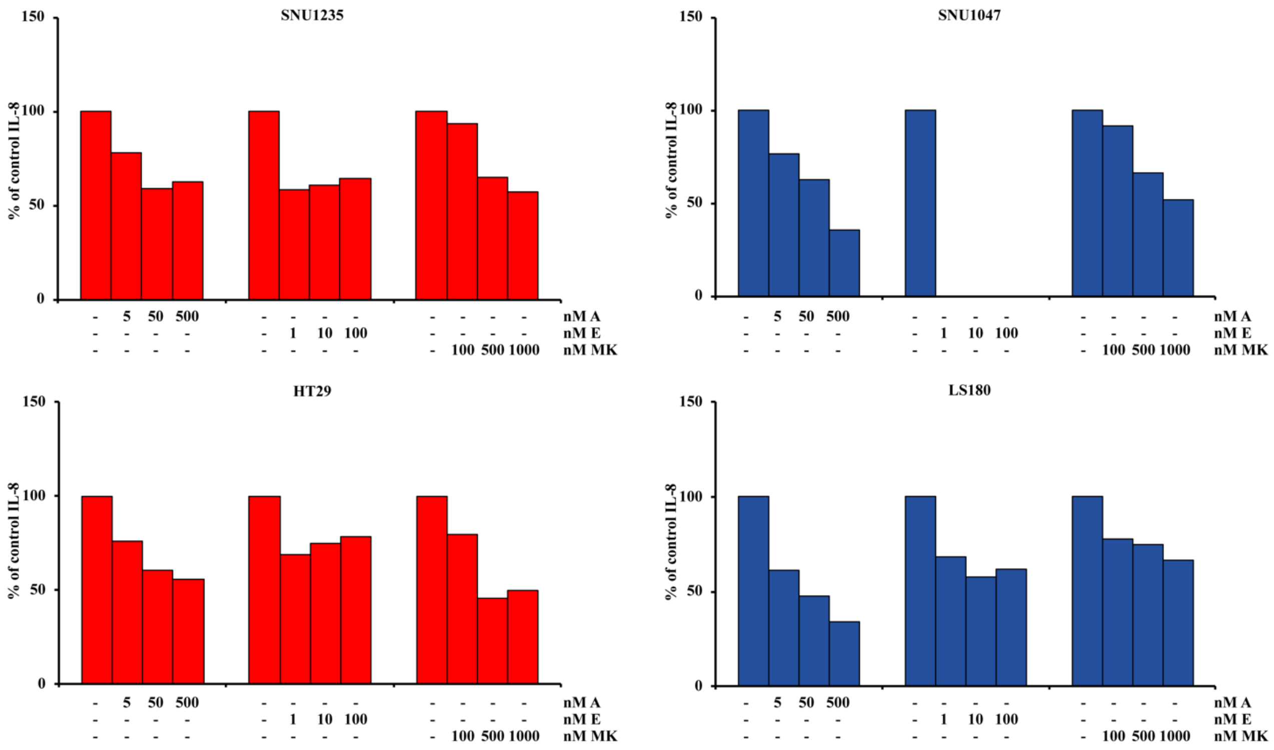


**Effects of PI3K/mTOR signaling inhibitors on IL-8 expression.** SNU1235, SNU1047, HT29 and LS180 cell lines were treated with increasing concentration of alpelisib (A), everolimus (E) or MK-2206 (MK), as indicated. IL-8 expression was measured after 24 hours of treatment, by IL-8 ELISA assay; the percentage of IL-8 was obtained from pg/mL assuming the levels in control cells as 100%. Results of a representative experiment out of three independent experiments performed are shown.

## G. Supplementary Fig. 7

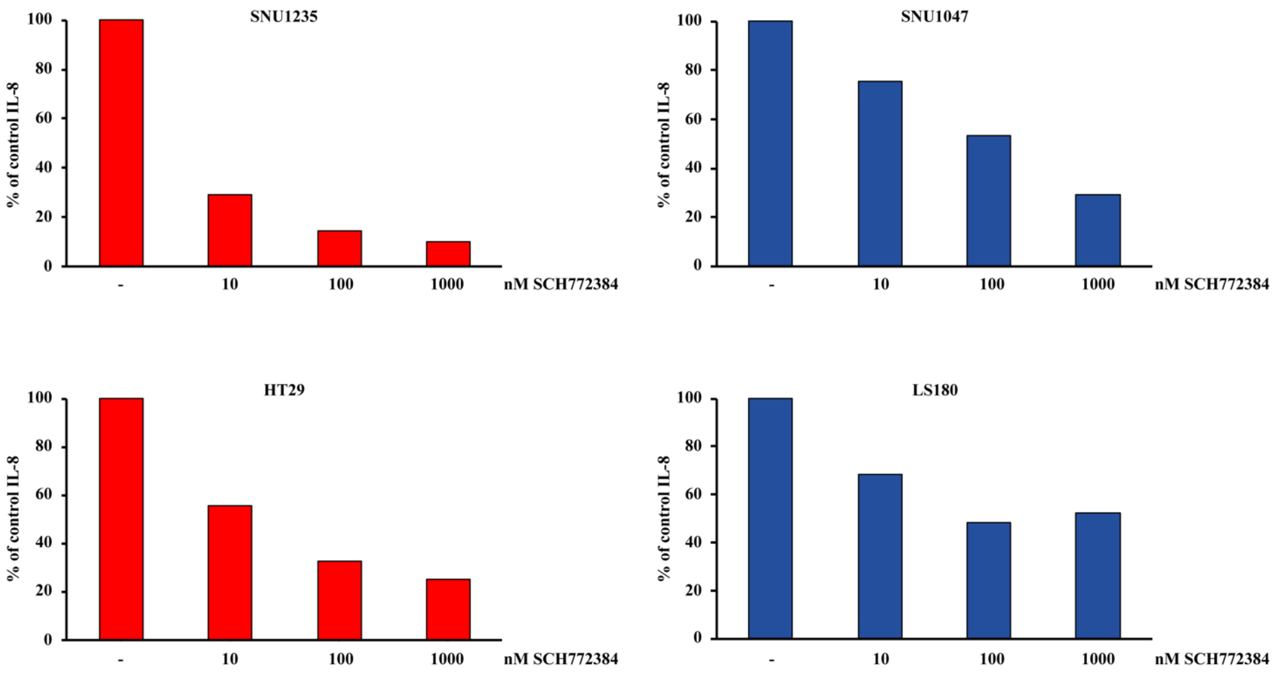


**Effects of ERK1/2 inhibition on IL-8 expression.** SNU1235, SNU1047, HT29 and LS180 cell lines were treated with increasing concentration of SCH772384, as indicated. IL-8 expression was measured after 24 hours of treatment, by IL-8 ELISA assay; the percentage of IL-8 was obtained from pg/mL assuming the levels in control cells as 100%. Results of a representative experiment out of three independent experiments performed are shown.

# H. Supplementary Fig. 8

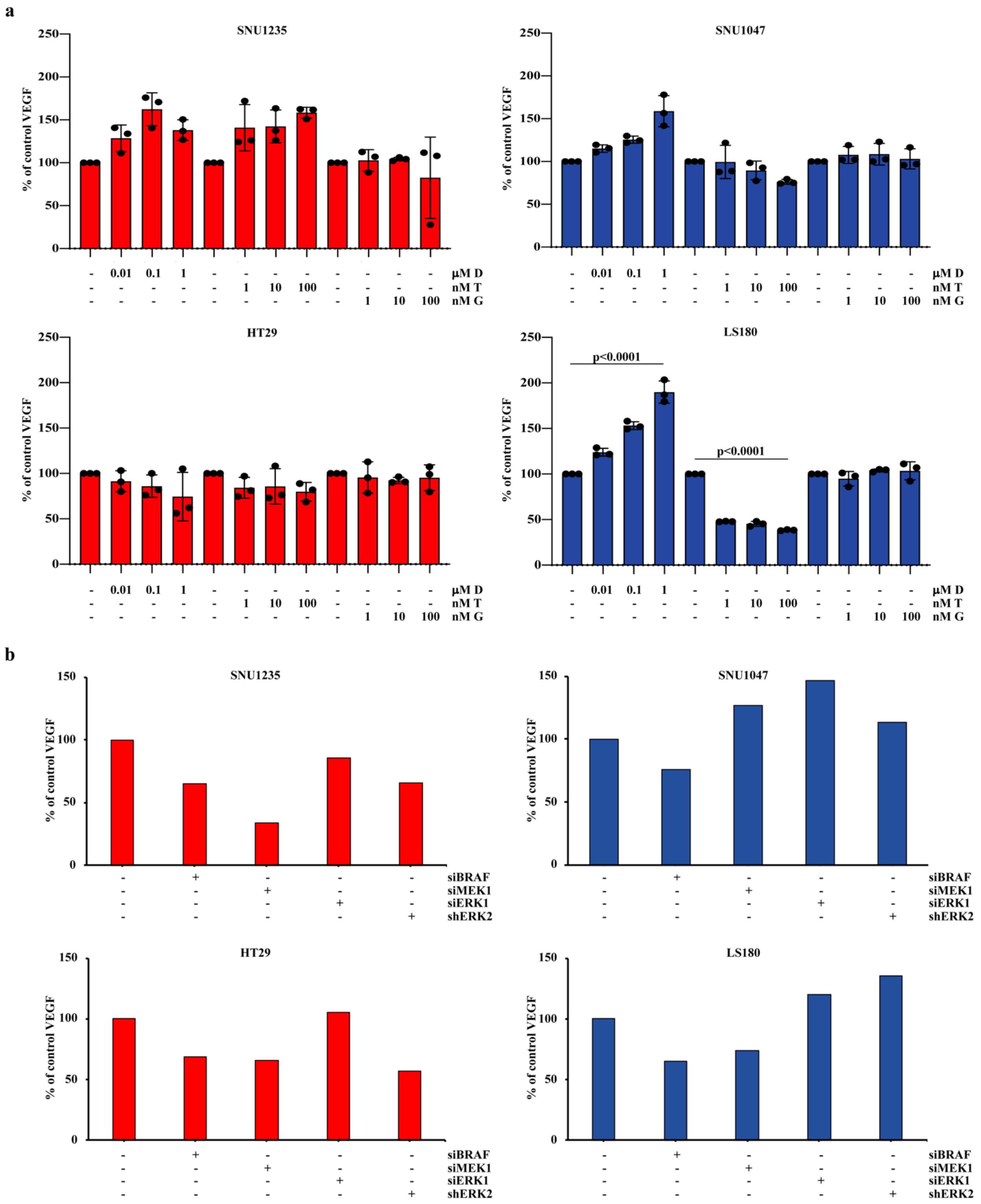


**Analysis of MAPK elements in affecting VEGF expression. a.** SNU1235, SNU1047, HT29 and LS180 cell lines were treated with increasing concentration of dabrafenib (D), trametinib (T) and gedatolisib (G), as indicated. VEGF expression was measured after 24 hours of treatment, by VEGF

ELISA assay; the percentage of VEGF was obtained from pg/mL assuming the levels in control cells as 100%. Results represent the average of three independent experiments. **b.** BRAF, MEK, ERK1 and ERK2 were knocked down by transient transfection of RNA interference for 24 hours, according to the manufacture's protocol. VEGF was measured after 24 hours of serum-free media; VEGF levels were measured as pg/mL and results are expressed as % of untreated control levels. Results of a representative experiment out of three independent experiments performed are shown..

Mean comparison of more than two groups was made by ANOVA and p-values indicate statistically significant differences ($p < 0.05$) (**a**).

I. Supplementary Fig. 9

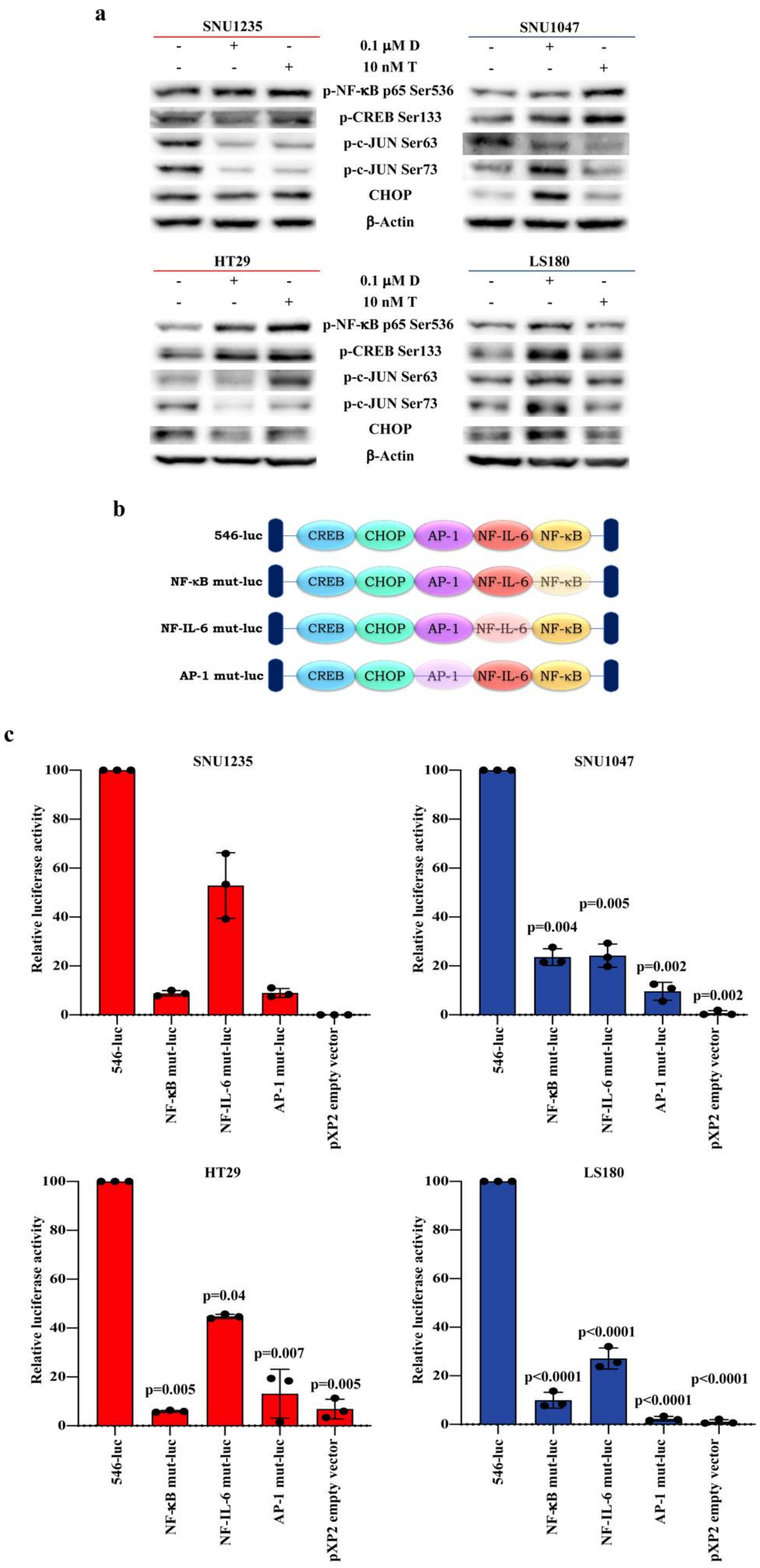

**Analysis of promoter elements in IL-8 expression. a.** SNU1235, SNU1047, HT29 and LS180 cells were treated with fixed doses of dabrafenib (D) and trametinib (T) for 24 hours, as indicated. Cells were lysed and analyzed by WB using antibodies specific for the indicated proteins. WB with β-Actin specific antibody is shown as protein loading and blotting control. **b.** Transcription factor binding regions in the IL-8 gene promoter retained in the different luciferase reporter plasmids. **c.** Cell lines were co-transfected with 100 ng luc-reporter vector and 10 ng pRL-TK. Cells were harvested for luc assay 24 hours post-transfection. pXP2 empty vector plasmid was used as a control. Results represent the average of three independent experiments. p-values indicate statistically significant differences ($p < 0.05$ by 2-tailed Student's t test) for the comparison between each reporter construct and the full promoter sequence (546-luc).

# J. Supplementary Fig. 10

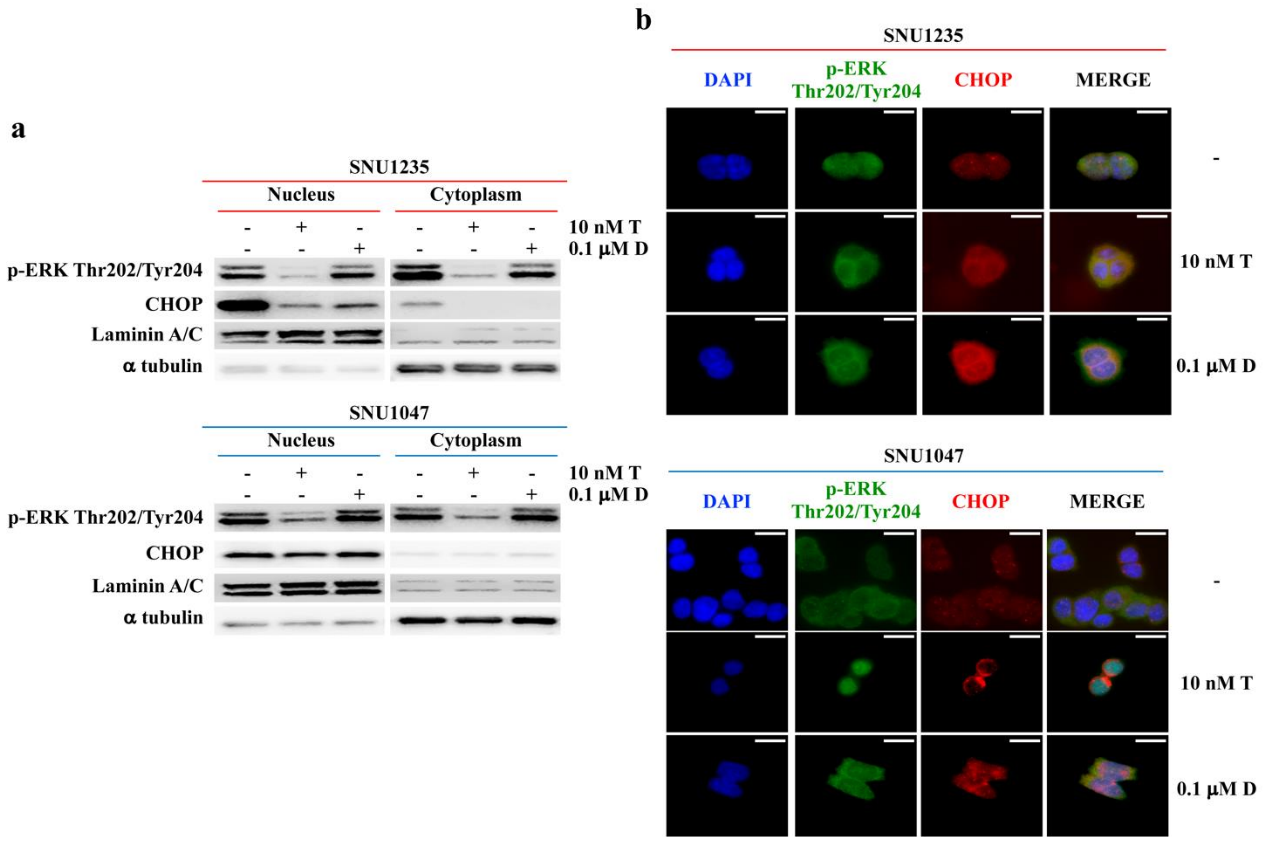


**CHOP subcellular localization after trametinib and dabrafenib treatment. a.** SNU1235 and SNU1047 cell lines were treated with fixed doses of trametinib (T) and dabrafenib (D) for 24 hours, as indicated. Cytoplasmic and nuclear fractions of cell lines were isolated; molecular effects were analyzed by WB using specific antibodies (Laminin A/C and α tubulin are shown as protein loading and blotting control for the nuclear and cytoplasmic compartments, respectively). **b.** Direct immunofluorescence analysis of the localization of CHOP (red) and p-ERK Thr202/Tyr204 (green) protein in the cytoplasm and nucleus (blue) of SNU1235 and SNU1047 cells, after T and D treatment. Results of a representative experiment out of three independent experiments performed are shown. Scale bars 10 µm.

# K. Supplementary Fig. 11, Original images for Western Blot

Figure 3a

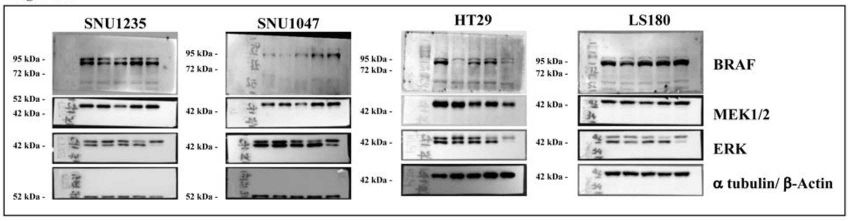


Supplementary Fig. 1

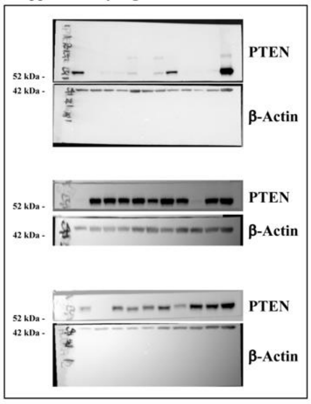


Figure 5a

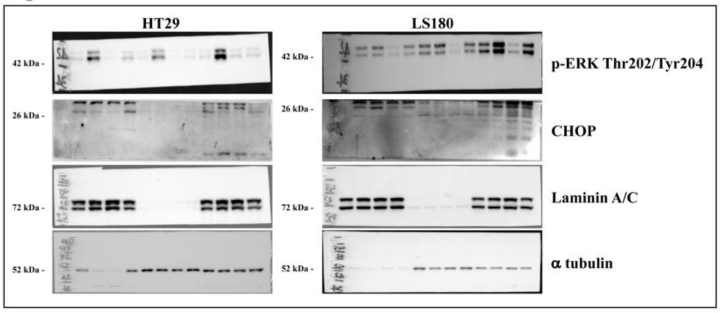


Figure 6

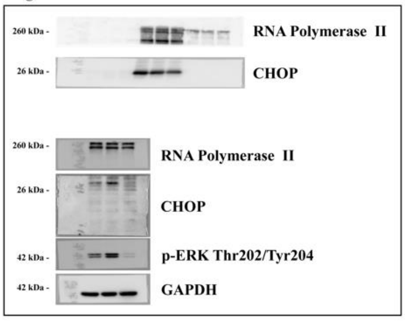


Supplementary Fig. 9a

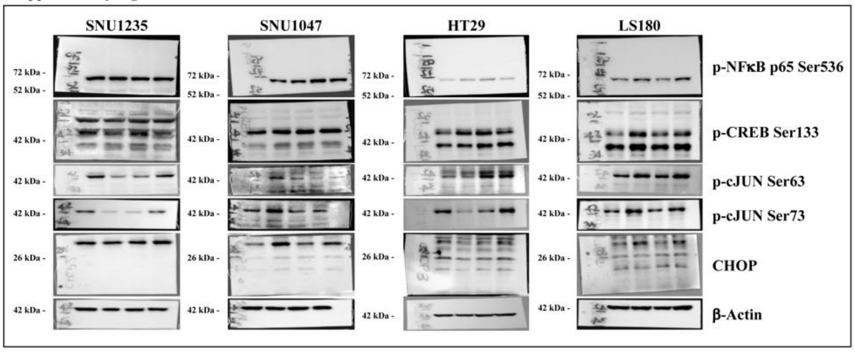


Supplementary Fig. 10a

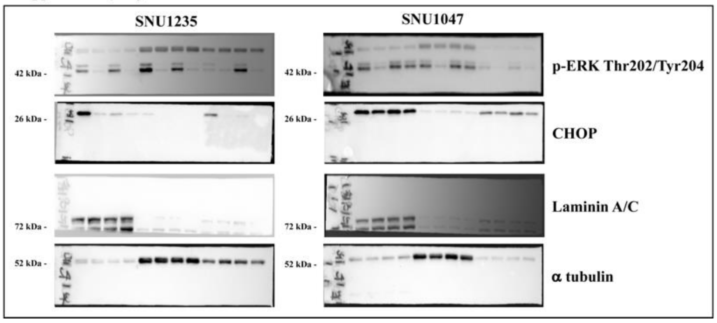

L. Supplementary Fig. 12

```
Query  836   TTTAAATGTATATTAAAATTAAATTATTTTAAAGATCAAAGAAAACTTTCGTCATACTCC  895
             ||||||||||||||||||||||||||||||||||||||||||||||||||||||||||||
Sbjct  8     TTTAAATGTATATTAAAATTAAATTATTTTAAAGATCAAAGAAAACTTTCGTCATACTCC  67

Query  896   GTATTTGATAAGGAACAAATAGGAAGTGTGATGACTCAGGTTTGCCCTGAGGGGATGGC  955
             |||||||||||||||||||||||||        ||||||||||||||||||||||||||
Sbjct  68    GTATTTGATAAGGAACAAATAGGAA--------ACTCAGGTTTGCCCTGAGGGGATGGC  119

Query  956   CATCAGTTGCAAATCGTGGAATTTCCTCTGACATAATGAAAAGATGAGGGTGCATAAGTT  1015
             ||||||||||||||||||||||||||||||||||||||||||||||||||||||||||||
Sbjct  120   CATCAGTTGCAAATCGTGGAATTTCCTCTGACATAATGAAAAGATGAGGGTGCATAAGTT  179

Query  1016  CTCTAGTAGGGTGATGATATAAAAAGCCACCGGAGCACTCCATAAGGCACAAACTTTCAG  1075
             ||||||||||||||||||||||||||||||||||||||||||||||||||||||||||||
Sbjct  180   CTCTAGTAGGGTGATGATATAAAAAGCCACCGGAGCACTCCATAAGGCACAAACTTTCAG  239

Query  1076  AGACAGCAGAGCACACAAGCTT  1097
             ||||||||||||||||||||||
Sbjct  240   AGACAGCAGAGCACACAAGCTT  261
```

**CHOP mut-luc vector sequence.** 546-luc vector was deleted of CHOP binding site region by using QuikChange II XL Site-Directed Mutagenesis Kit. The lack of CHOP region was confirmed by the gene sequencing of both 546-luc vector (upper nucleotide string) and CHOP mut-luc vector (bottom nucleotide string) and the comparison between the sequences.

## M. Supplementary Table 1.

**Additional information for NGS relevant mutations**

| Cell lines | Gene | AF% | AA | Nucl | Cov Mut | Exon | Cosmic | Path Level |
|---|---|---|---|---|---|---|---|---|
| **SNU1544** | PI3KCA | 51 | H1047R | 3140 A>G | 953 | 21 | 775 | I |
| **MDST8** | BRAF | 80.9 | V600K | 1798_1799 GT>AA | n.a. | 15 | 473 | |
| **LIM2537** | BRAF | 64.94 | V600E | 1799 T>A | 1985 | 15 | 476 | |
| **LIM2412** | BRAF | 68.12 | V600E | 1799 T>A | 1910 | 15 | 476 | |
| **LS411N** | BRAF | 69.39 | V600E | 1799 T>A | 539 | 15 | 476 | |
| **LS411N** | PTEN | 50.07 | C105fs | 313delT | 1428 | 5 | | |
| **SNUC4** | PTEN | 47.55 | F241S | 722 T>C | 2000 | 7 | | |
| **SNUC4** | PTEN | 29.53 | V290* | 867delA | 1981 | 8 | | |
| **SNUC4** | PI3KCA | 49,27 | V71I | 211 G>A | 1999 | 2 | | |
| **SNUC4** | PI3KCA | 24.99 | E545G | 1634 A>G | 1985 | 10 | | |
| **SNUC5** | BRAF | 51.41 | V600E | 1799 T>A | n.a. | 15 | 476 | |
| **SNUC5** | PI3KCA | 55.29 | H1047R | 3140 A>G | 293 | 21 | | |
| **SNU1040** | PTEN | 70.46 | R335* | 1003 C>T | 1581 | 8 | | |
| **SNU1040** | PTEN | 31.23 | T232A | 694 A>G | 1998 | 7 | | |
| **SNU1047** | PTEN | 100 | K267fs | 800delA | n.a. | 7 | 5809 | |
| **SNU1235** | BRAF | 67 | V600E | 1799 T>A | n.a. | 15 | 476 | |
| **SNU1235** | PTEN | 99.8 | R130* | 388 C>T | n.a. | 5 | 5152 | |
| **KM12C** | PTEN | 51.2 | G129* | 385 G>T | 1956 | 5 | | |
| **VACO432** | BRAF | 49.42 | V600E | 1799 T>A | 690 | 15 | 476 | |
| **VACO432** | PI3KCA | 20.17 | H1047R | 3140 A>G | 872 | 21 | 775 | I |
| **HT29** | BRAF | 23.6 | V600E | 1799 T>A | n.a. | 15 | 476 | |
| **LS180** | KRAS | 50.6 | G12D | 35 G>A | n.a. | 2 | 521 | |
| **LS180** | PI3KCA | 53.3 | H1047R | 3140 A>G | n.a. | 21 | 775 | |
| **SW620** | KRAS | 99 | G12V | 35 G>T | 1991 | 2 | 520 | I |
| **SW480** | KRAS | 99.6 | G12V | 35 G>T | 1280 | 2 | 520 | I |
| **COLO205** | BRAF | 66.09 | V600E | 1799 T>A | 864 | 15 | 476 | |
| **RKO** | BRAF | 68.18 | V600E | 1799 T>A | 1546 | 15 | 476 | |
| **RKO** | PI3KCA | 56.78 | I391M | 1173 A>G | 752 | 7 | | |
| **RKO** | PI3KCA | 53.19 | H1047R | 3140 A>G | 1286 | 21 | | |
| **COGA3** | KRAS | 47.71 | G13D | 38 G>A | 1989 | 2 | 532 | I |
| **COGA3** | PTEN | 49.4 | R173H | 518 G>A | 1996 | 6 | | |
| **HCT116** | KRAS | 54.06 | G13D | 38 G>A | 862 | 2 | 532 | I |
| **HCT116** | PI3KCA | 49.11 | H1047R | 3140 A>G | 733 | 21 | 775 | I |
| **HCT116 PTEN$^{-/-}$** | KRAS | 50.44 | G13D | 38 G>A | 1929 | 2 | 532 | I |
| **HCT116 PTEN$^{-/-}$** | PI3KCA | 48.77 | H1047R | 3140 A>G | 1747 | 21 | 775 | I |
| **HKE-3** | PI3KCA | 49.85 | H1047R | 3140 A>G | 2000 | 21 | 775 | I |
| **HK2-6** | KRAS | 45.48 | G12C | 34 G>T | 1990 | 2 | | |

| | | | | | | | | |
|---|---|---|---|---|---|---|---|---|
| **HK2-6** | KRAS | 53.24 | G13D | 38 G>A | 1991 | 2 | 532 | I |
| **HK2-6** | PI3KCA | 48.4 | H1047R | 3140 A>G | 2000 | 17 | 775 | I |
| **DLD-1** | KRAS | 50 | G13D | 38 G>A | 1904 | 2 | 532 | I |
| **DLD-1** | PI3KCA | 52 | E545K | 1633 G>A | 1258 | 10 | 763 | I |
| **DLD-1** | PI3KCA | 52 | D549N | 1645 G>A | 1228 | 10 | | |
| **DKO-1** | KRAS | 13.65 | G12C | 34 G>T | 1990 | 2 | | |
| **DKO-1** | KRAS | 54.61 | G13D | 38 G>A | 1991 | 2 | 532 | I |
| **DKO-1** | PI3KCA | 53.01 | E545K | 1633 G>A | 1941 | 10 | | |
| **DKO-1** | PI3KCA | 51.97 | D549N | 1645 G>A | 1901 | 10 | | |
| **DKO-4** | PI3KCA | 51.42 | E545K | 1633 G>A | 1941 | 10 | | |
| **DKO-4** | PI3KCA | 51.2 | D549N | 1645 G>A | 623 | 10 | | |

AF, allele frequency; AA, amino acid; Nucl, nucleotide; Cov Mut, coverage mutation; n.a., not available.
